# Molecular characterization of porcine epidemic diarrhoea virus (PEDV) in Poland reveals the presence of swine enteric coronavirus (SeCoV) sequence in S gene

**DOI:** 10.1371/journal.pone.0258318

**Published:** 2021-10-29

**Authors:** Marta Antas, Monika Olech, Anna Szczotka-Bochniarz

**Affiliations:** 1 Department of Swine Diseases, National Veterinary Research Institute, Puławy, Poland; 2 Department of Biochemistry, National Veterinary Research Institute, Puławy, Poland; University of Nicolaus Copernicus in Torun, POLAND

## Abstract

Porcine epidemic diarrhoea (PED) is a highly contagious enteric viral disease of pigs with a high morbidity and mortality rate, which ultimately results in huge economic losses in the pig production sector. The etiological agent of this disease is the porcine epidemic diarrhoea virus (PEDV) which is an enveloped, positive single-stranded RNA virus. The aim of this study was to perform molecular characterization of PEDV to identify the strains circulating in Poland. In this study, 662 faecal samples from 2015 to 2021 were tested with reverse transcription quantitative real-time PCR (RT-qPCR) and the results showed that 3.8% of the tested samples revealed a positive result for PEDV. A phylogenetic analysis of the complete genome and complete S gene sequences showed that Polish PEDV strains belonged to the G1b (S-INDEL) subgroup and were closely related to the European PEDV strains isolated from 2014 to 2019. Furthermore, RDP4 analysis revealed that the Polish PEDV strains harboured a recombinant fragment of ~400 nt in the 5’ end of S gene with PEDV and swine enteric coronavirus (SeCoV) being the major and minor parents, respectively. Antigenic analysis showed that the aa sequences of neutralizing epitopes were conserved among the Polish PEDV strains. Only one strain, #0100/5P, had a unique substitution in the COE epitope. However, Polish PEDV strains showed several substitutions, especially in the COE antigen, as compared to the classical strain CV777. To the best of our knowledge, this is the first report concerning the molecular characterization of porcine epidemic diarrhoea virus strains, as well as the first phylogenetic analysis for PEDV in Poland.

## Introduction

Porcine epidemic diarrhoea (PED), an acute and highly contagious enteric disease of pigs is characterized by watery diarrhoea, vomiting, dehydration and weight loss. The disease affects pigs of all ages but the most susceptible are neonatal piglets among which mortality can reach 100%, resulting in substantial economic losses [1–3]. The etiological agent of this disease is porcine epidemic diarrhoea virus (PEDV), which is an enveloped positive single-stranded RNA virus belonging to the *Coronaviridae* family [1, 4, 5]. The genome of PEDV is approximately 28 kb in size and contains seven open reading frames (ORF1a, ORF1b and ORF2-6) encoding four structural proteins (spike (S), envelope (E), membrane (M), nucleocapsid (N)), two non-structural proteins (pp1a and pp1ab) and one accessory protein encoded by ORF3 [6, 7]. Among these proteins, the main interest is focused on the S protein which in the presence of host proteolytic trypsin is cleaved into two subunits, S1 and S2 which are responsible for cellular receptor binding and fusion activity, respectively. Moreover, the S protein is also the main glycoprotein, which induces neutralizing antibody production [8, 9]. To date, four neutralizing epitopes have been characterized in the S protein. Three of them are located in domain S1 (core neutralizing epitope (COE) residues 499–638, SS2 residues 746–755 and SS6 residues 764–771) and the fourth is in domain S2 (2C10 residues 1368–1374). In addition, the S gene exhibits a high degree of genetic diversity, especially in the S1 subunit, which is also related to the attenuation of PEDV virulence *in vivo* and growth adaptation *in vitro* [3, 9, 10].

Based on the nucleotide sequence of the S gene, two main genogroups (G) of PEDV have been described, G1 (S-INDEL) which has insertions and deletions (INDEL) in the S1 subunits of the S protein and G2 (non-S INDEL). Genogroups G1 and G2 can be further divided into two subgroups G1a (classical strains), G1b (S-INDEL strains) and G2a (variant strain), G2b (recombinant strains), respectively [11–14]. The classical PEDV (strain CV777), grouped as G1a, was first recognized as the causative agent of severe swine enteric disease in the 1970s in England [2, 15]. Thereafter the virus spread to several countries in Europe and from Europe to Asia, resulting in large outbreaks with considerable losses to the pig industry [4, 16]. Since 2010, a new highly virulent PEDV strain belonging to G2 spread throughout the Unites States, and other countries in North, Central and South America [17, 18]. In addition, a second PEDV strain OH851 with a lower level of virulence, belonging to the G1b group, have been identified in the United States [19], South Korea [20] and Japan [21]. The circulation of these mild PEDV strains have also been reported in several European countries since 2014 [2, 3].

In Poland, the clinical symptoms of PED with acute diarrhoea and high mortality in pigs have been observed, but until 2015 no studies had been performed to confirm the presence of PEDV. In 2015–2017 the presence of specific antibodies against PEDV was identified in several farms in Poland but up to the present time there has been a notable absence of information concerning the genetic characteristics of PEDV strains circulating in Poland. Thus, the aim of this study was to perform molecular characterization of PEDV to identify the strains circulating in Poland. In this study, for the first time, we described sequences of Polish PEDV strains and performed phylogenetic analysis to determine the relationship between Polish PEDV strains and others, circulating worldwide.

## Materials and methods

### Sample collection and preparation

A total of 662 faecal samples were collected from pigs (sows, boars, finishers and nursing piglets) with clinical signs suggestive of PEDV infection from 63 commercial herds located in 10 voivodeships of Poland (Wielkopolskie, Mazowieckie, Świętokrzyskie, Pomorskie, Warmińsko-Mazurskie, Lubelskie, Podlaskie, Kujawsko-Pomorskie, Dolnosląskie, Zachodniopomorskie) in 2015–2021. The samples were diluted 1:10 (v/v) with phosphate-buffered saline (PBS) (137 mM NaCl, 2.7 mM KCl, 10.1 mM Na_2_HPO4 and 1.8 mM KH_2_PO4), homogenized by vortex mixing and centrifuged for 8 min at 6,000 g at 4°C. The clarified supernatants were collected and stored at -80°C for RNA extraction. Faecal samples were non-invasively collected from pigs immediately after defecation by qualified veterinarians as a part of their routine veterinary supervision, therefore no ethics committee approval was required [22]. Verbal informed consent has been obtained from all owners prior to the collection of faecal samples from the pigs.

### RNA extraction and PEDV identification

The total of RNA was extracted from 140 μl of the collected supernatant using a QIAMP Viral RNA Mini Kit (Qiagen, Germany) according to the manufacturer’s protocol. Extracted viral RNA was subjected to triplex RT-qPCR using a VetMAXTM PEDV/TGEV/SDCoV Kit (Thermo Fisher Scientific, USA) according to the manufacturer’s recommendations. For each separate reverse transcription quantitative real-time polymerase chain reaction (RT-qPCR) run two positive controls (one for the RT-qPCR components and another for the RNA purification process) as well as one negative control (nuclease-free water, Ameresco, USA) were included. The RT-qPCR were run on the Mx3005P qPCR System and Aria MX (Agilent Technologies, USA) according to the manufacturer’s instructions. A positive control was used to set the cycle threshold (Ct) for evaluating the test results. A VetMAXTM PEDV/TGEV/SDCoV Kit was used to detect PEDV infection and exclude a potential PEDV- transmissible gastroenteritis virus (TGEV) and PEDV- porcine deltacoronavirus (PDCoV) co-infection [23].

### NGS and Sanger sequencing

Total RNA extraction of the PEDV positive samples were subjected to Next Generation Sequencing (NGS) using Illumina’s sequencing-by-synthesis (SBS) technology. For samples which could not be sequenced by NGS, sequences of the spike gene (S gene) were obtained by sequencing in both directions using the Sanger methodology. Four overlapping fragments were amplified with the primers described in Table 1 and the OneStep RT-PCR Kit (Qiagen, Germany). The reaction was conducted under the following conditions: 50°C for 30 min, 95°C for 15 min, 40 cycles at 95°C for 40 s, 55–58°C for 1 min and 72°C for 1 min, followed by a final extension step at 72°C for 10 min. The reverse transcription polymerase chain reaction (RT-PCR) products were visualized under ultraviolet (UV) light after electrophoresis in a 1.5% agarose gel, containing SimplySafe (EURx, Poland) in 1 x Tris-acetate-EDTA (TAE) Buffer (40 mM Tris/acetate buffer and 1 mM ethylenediamine tetraacetic acid (EDTA), pH 8.0). The RT-PCR products were purified using NucleoSpin Gel and PCR Clean-up (Macherey-Nagel, Germany) and sequenced on a 3730xl DNA Analyser (Applied Biosystems, USA) using a Big Dye Terminator v3.1 Cycle Sequencing kit (Applied Biosystems, USA). The NGS and Sanger sequencing was performed by a commercial company (Genomed S.A., Warsaw, Poland). All of the novel sequences reported in this study were submitted to the Gen-Bank database under accession numbers: MZ216018-MZ216031 and MZ268115, MZ313556-MZ313557, MZ325484-MZ325487.

**Table 1:** Primers used in the amplification of the S-gene.

Name	Sequence 5’ -3’	Product size	Product name	Source
**S-F1**	TGCTAGTGCGTAATAATGAC	1,349	I	Huang et al., 2013 [24]
**ED-S1R**	CGTCAGTGCCATGACCAGTG			de Nova et al., 2020 [25]
**ED-2F**	GGGAAATTGTCATCACCAAG	1,289	II	de Nova et al., 2020 [25]
**PEDV-S1R**	CTGGGTGAGTAATTGTTTACAACG			Chen et al., 2014 [26]
**ED-S3F**	AGTACTAGGGAGTTGCCTGG	1,216	III	de Nova et al., 2020 [25]
**ED-S3R**	AACCATAACGCTGAGATTGC			de Nova et al., 2020 [25]
**ED-S4F**	TTGAACACTGTGGCTCATGC	1,128	IV	de Nova et al., 2020 [25]
**S-R1**	CATCTTTGACAACTGTGT			Huang et al., 2013 [24]

### Sequence analysis

The quality of the NGS reads was checked with FastQC software [27]. After that, Cutadapt (ver. 1.16) [28] was used to remove adapters, reads shorter than 25 base pairs. Processed reads were mapped to the reference genome SLOreBAS-1/2015 (KY019623) with BWA (ver. 0.7.15-r1140) [29]. Mapped reads were filtered using Samtools (version 1.6) [30], which were then assembled *de novo* using Spades (version 3.11.1) [31]. The S-gene sequences obtained by Sanger sequencing were edited and assembled using Geneious Pro 5.3 software (Biomatters Ltd, New Zealand). Manual rearrangements of the alignments, including gap exclusion and length adjustment were carried out to achieve optimal results. The evolutionary relationship between the sequences obtained in this study and other reference sequences deposited in GenBank were investigated by constructing phylogenetic trees from multiple alignments using ClustalW. Unrooted phylogenetic trees based on whole genome sequences and the S gene sequences were constructed using the neighbor joining (NJ) method which in turn used the maximum composite likelihood model. Nonparametric Bootstrap analysis with 1000 iterations was used to evaluate the robustness of the evolutionary relationships. Alignment and NJ tree building were performed using MEGA software version 6.06 [32]. The sequence percentage identity (percentage of bases/residues which are identical) was derived using Geneious software.

### Analysis of recombination

In order to detect possible recombination events, the Recombination Detection Program version 4 (RDP4) with the default setting was used [33]. The software used seven primary exploratory recombination signal detection methods, RDP [34], GENECONV [35], BootScan [36], MaxChi [37], Chimaera [38], SiScan [39] and 3Seq [40]. The beginning and end breakpoints of the potential recombinant sequences were also defined by the RDP4 software. Putative recombinant events were considered to be significant when P ⩽ 0.01 was observed for the same event using four or more algorithms.

## Results

### Detection of PEDV using molecular techniques

In this study, of the 662 processed samples from 63 herds, 25 (3.8%) samples originating from 4 herds (6.3%) were found to be positive for PEDV through the use of RT-qPCR (Table 2). These positive samples originated from three distinct voivodeships: Kujawsko-Pomorskie, Dolnośląskie and Wielkopolskie. The possibility of a PEDV-TGEV and PEDV-PDCoV co-infection was ruled out as neither TGEV nor PDCoV sequences were detected in any of samples tested. All 25 samples were subjected to NGS sequencing but only seven samples passed the quality assurance (QC) test necessary for successful sequencing. NGS reads of all seven samples (#44176/1, #6220, #6706/2, #25364/2, #0100/4T, #0100/1L, #0100/2M) were successfully mapped against the complete genome sequence of the PEDV reference strain SLOreBAS-1/2015 (KY019623). The obtained PEDV nucleotide sequences ranged from 28,005 to 28,048 nucleotides in length.

**Table 2:** Characteristics of the Polish porcine epidemic diarrhoea virus (PEDV) isolates selected for genetic analysis.

No	Isolate	Herd	Acc. No	Sample origin	Collection date	RT-qPCR result	Sequence type
1	44176/1	1	MZ325487	Radojewice	2016.12.27	+	Complete genome
2	44176/2		MZ216019	Radojewice	2016.12.27	+	Complete S-gene
3	44176/3		-	Radojewice	2016.12.27	+	-
4	44176/4		-	Radojewice	2016.12.27	+	-
5	0100/3P		MZ216023	Radojewice	2017.01.03	+	Partial S-gene (II)
6	0100/4P		MZ216024	Radojewice	2017.01.03	+	Partial S-gene (I, IV)
			MZ216025				
7	0100/5P		MZ216018	Radojewice	2017.01.03	+	Complete S-gene
8	0100/1W		-	Radojewice	2017.01.03	+	-
9	0100/4W		-	Radojewice	2017.01.03	+	-
10	0100/5W		-	Radojewice	2017.01.03	+	-
11	0100/1T		-	Radojewice	2017.01.03	+	-
12	0100/2T		-	Radojewice	2017.01.03	+	-
13	0100/3T		MZ216022	Radojewice	2017.01.03	+	Complete S-gene
14	0100/4T		MZ313556	Radojewice	2017.01.03	+	Complete genome
15	0100/4M		MZ216026	Radojewice	2017.01.03	+	Partial S-gene (III, IV)
			MZ216027				
16	0100/5M		-	Radojewice	2017.01.03	+	-
17	0100/1L		MZ325484	Radojewice	2017.01.03	+	Complete genome
18	0100/3L		MZ216028	Radojewice	2017.01.03	+	Partial S-gene (III)
19	0100/5T		MZ216029	Radojewice	2017.01.03	+	Partial S-gene (I, II, IV)
			MZ216030				
			MZ216031				
20	0100/2M		MZ325485	Radojewice	2017.01.03	+	Complete genome
21	6220	2	MZ313557	Dobroszyce	2016.04.01	+	Complete genome
22	6706/1	3	MZ216020	Jutrosin	2016.04.07	+	Compete S-gene
23	6706/2		MZ325486	Jutrosin	2016.04.07	+	Complete genome
24	25364/1	4	MZ216021	Dobrzyca	2015.10.12	+	Complete S-gene
25	25364/2		MZ268115	Dobrzyca	2015.10.12	+	Complete genome

No- number.

Acc. No-Accession number.

The RNA of the remaining 18 samples, which could not be sequenced by NGS, was used for the amplification of the S gene. In the case of 5 samples (#44176/2, #6706/1, #25364/1, #0100/5P, #0100/4T) all 4 overlapping fragments were successfully amplified and complete S gene sequences were obtained. For 5 other samples only partial S gene sequences were obtained. Fragment I (see Tables 1 and 2) was successfully amplified and sequenced for sample #0100/4P and sample #0100/5T while fragment II was successfully amplified and sequenced for sample #0100/3P and sample #0100/5T. Fragments III and IV were successfully obtained for samples #0100/4M, #01003L and #01004P, #0100/4M, #0100/5T, respectively. None of the remaining 8 samples (#44176/3, #44176/4, #0100/1W, #0100/4W, #0100/5W, #0100/1T, #0100/2T, #0100/5M) produced positive amplification products.

### Phylogenetic analysis

**Fig 1:**
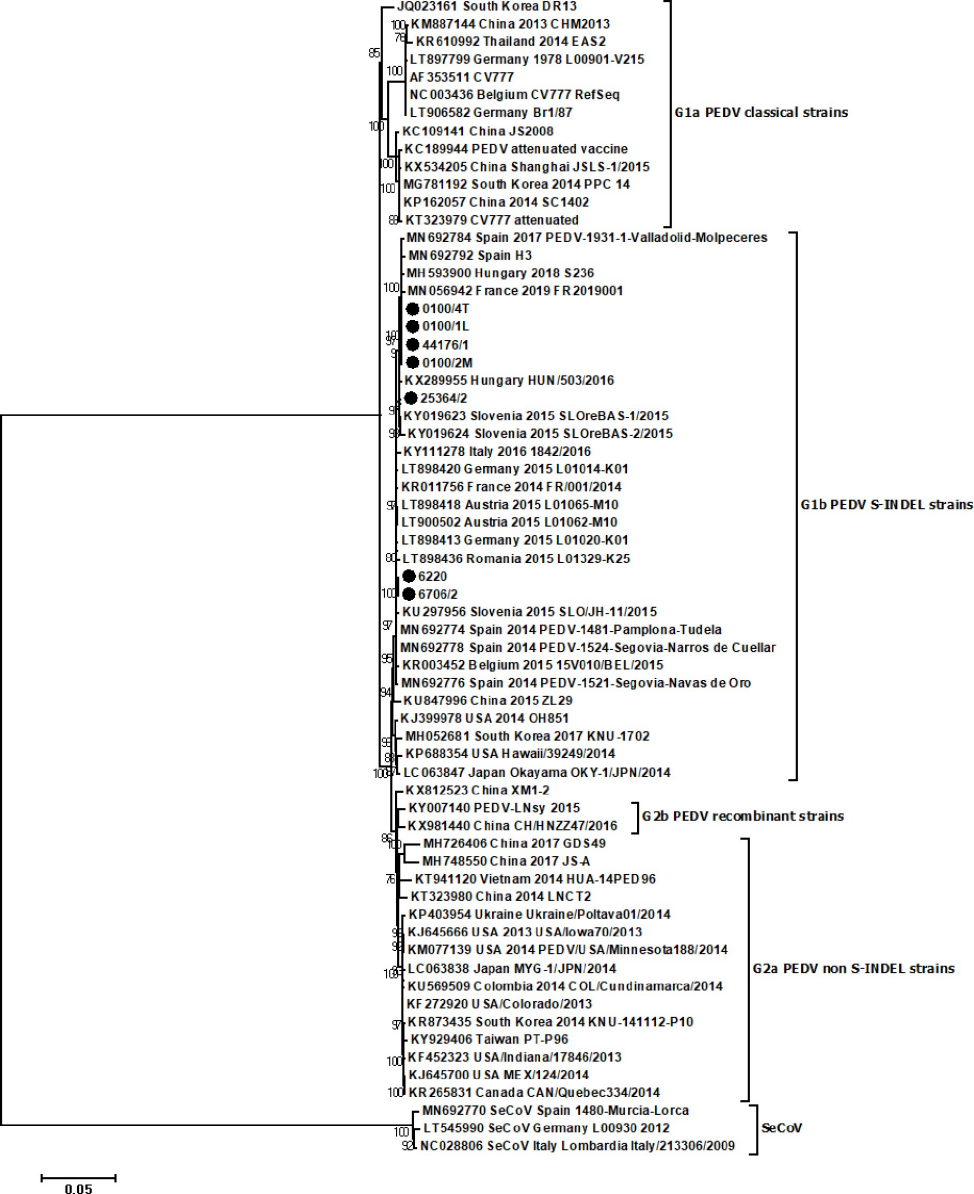
Phylogenetic relationship between the sequences of the PEDV Polish strains and the sequences of the reference strains obtained from GenBank. The phylogenetic tree was constructed on the basis of the complete genome sequences with MEGA6 software using the neighbor-joining method. Bootstrap values >70 are shown. The numbers of each branch represent the bootstrap value calculated using 1000 replicates. The scale bars indicate nucleotide substitutions per site. The S gene sequences from the PEDV isolates identified in this study are indicated with filled black circles.

The complete genome sequences of the 7 Polish strains obtained in this study were compared to other representative PEDV and swine enteric coronavirus (SeCoV) strains deposited in GenBank. The phylogenetic tree (Fig 1) showed that all Polish PEDV sequences were allocated, with a bootstrap value of 100%, within the G1b genogroup, together with sequences of European, Asian and American PEDV S-INDEL strains. They clustered in a branch which is clearly distinct from non-S-INDEL (G2 genogroup) isolates as well as from the original Asian and European strains included in the G1a genogroup and sequences of SeCoV isolates. Based on the whole genome sequence alignment, the Polish PEDV strains shared 99.5%-100% nucleotide identity with each other and 96.5%-100% and 61.2%-61.45% nucleotide identity with the representative PEDV and SeCoV strains analysed in this study. Polish PEDV strains showed the highest sequence identity value (99.6%-100%) with European PEDV strains (from Spain, France, Hungary, Italy, Slovenia, Germany, Romania, Belgium and Austria) isolated from 2014 to 2019.

**Fig 2:**
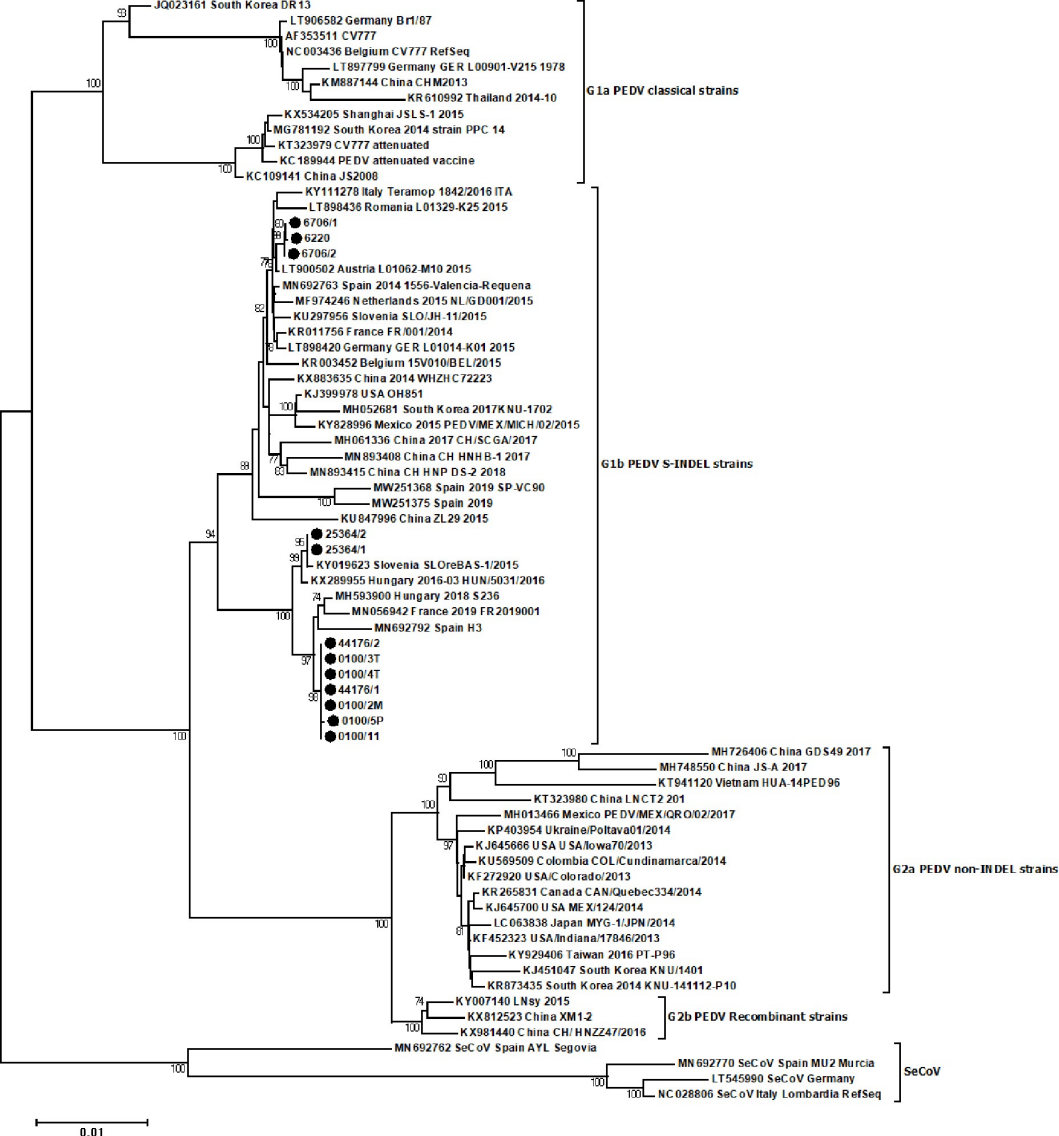
Phylogenetic relationship between the sequences of PEDV Polish strains and sequences of reference strains obtained from GenBank. The phylogenetic tree was constructed on the basis of the complete S gene sequences with MEGA6 software using the neighbor-joining method. Bootstrap values >70 are shown. The numbers of each branch represent the bootstrap value calculated using 1000 replicates. The scale bars indicate nucleotide substitutions per site. The S gene sequences from the PEDV isolates identified in this study are indicated with filled black circles.

The nucleotide identity varied between the full length S gene sequences of 12 Polish PEDV strains within a range of 98.4% to 100% while the sequence identities between the Polish PEDV isolates and representative PEDV and SeCoV strains varied from 94.0% to 100% and from 91.1% to 94.2%, respectively. The phylogenetic tree (Fig 2) based on the full length S gene sequences confirmed that Polish PEDV strains were allocated within G1b subgroup. This affiliation was supported by a high bootstrap value (94%). Three clusters within the G1b subgroup were identified from Polish PEDV strains on the basis of the S gene sequences. The first was formed by sequences of three Polish isolates (#6706/1, #6220 and #6706/2) which originated from 2016 together with sequences of European, Asian and American PEDV S-INDEL isolates. These Polish PEDV isolates showed the closest sequence identity with the PEDV sequence from Austria (LT900502), France (KR011756), Spain (MN692763), Germany (LT898420), Slovenia (KU297956) and the Netherlands (MF974246) (99.7%-99.9%). The second cluster included two Polish strains (#25364/1 and #25364/2) from 2015 which originated from one herd which showed the closest sequence identity with the sequence of the Hungarian strain #HUN/5031/2016 (KX289955) and Slovenian strain #SLOreBAS-1/2015 (KY019623) (99.9%-100%). Finally, the third cluster was formed by 7 sequences of Polish PEDV strains (#44176/2, #44176/1, #0100/3T, #0100/4T, #0100/2M, #0100/5P and #0100/1L) located together with the Hungarian, French and Spanish strains (99.3%-99.8% nucleotide identity). Furthermore, an additional analysis of the partial sequences of the S gene revealed that the Polish strains #0100/3P, #0100/4P, #0100/4M, #0100/3L and #0100/5T belonged to the third cluster within the G1b subgroup together with strains #44176/2, #44176/1, #0100/3T, #0100/4T, #0100/2M, #0100/5P and #0100/1L (S1–S4 Figs). It is worth mentioning that all of the isolates identified in this third cluster correspond to the isolates obtained from the same herd between 2016 and 2017.

### Deduced amino acid sequence analysis of neutralizing epitopes in the S protein

**Fig 3:**
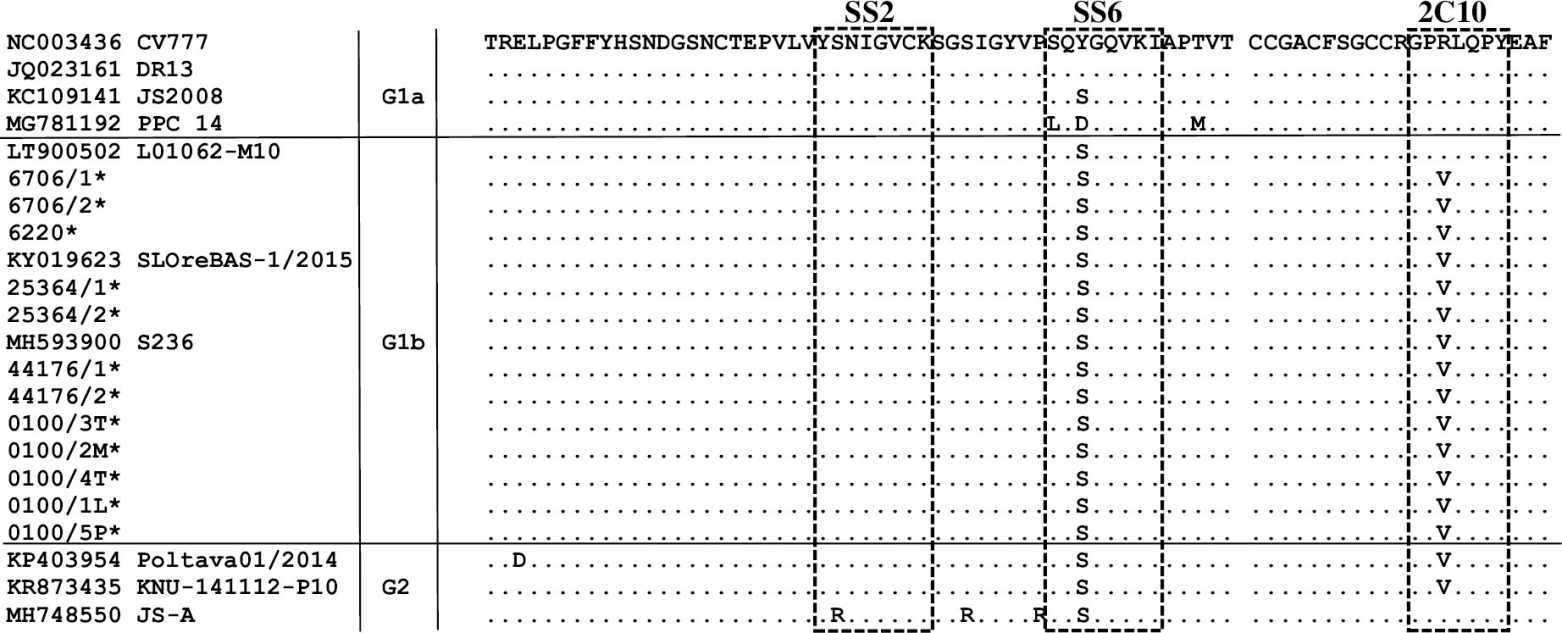
Amino acid alignment of the neutralizing epitope SS2, SS6 and 2C10 located in the S protein of the Polish PEDV strains and reference strains. The dots (.) represent amino acids that are identical. Alignment was constructed using the Clustal W method. Stars indicate the Polish PEDV sequences described in this study.

**Fig 4:**
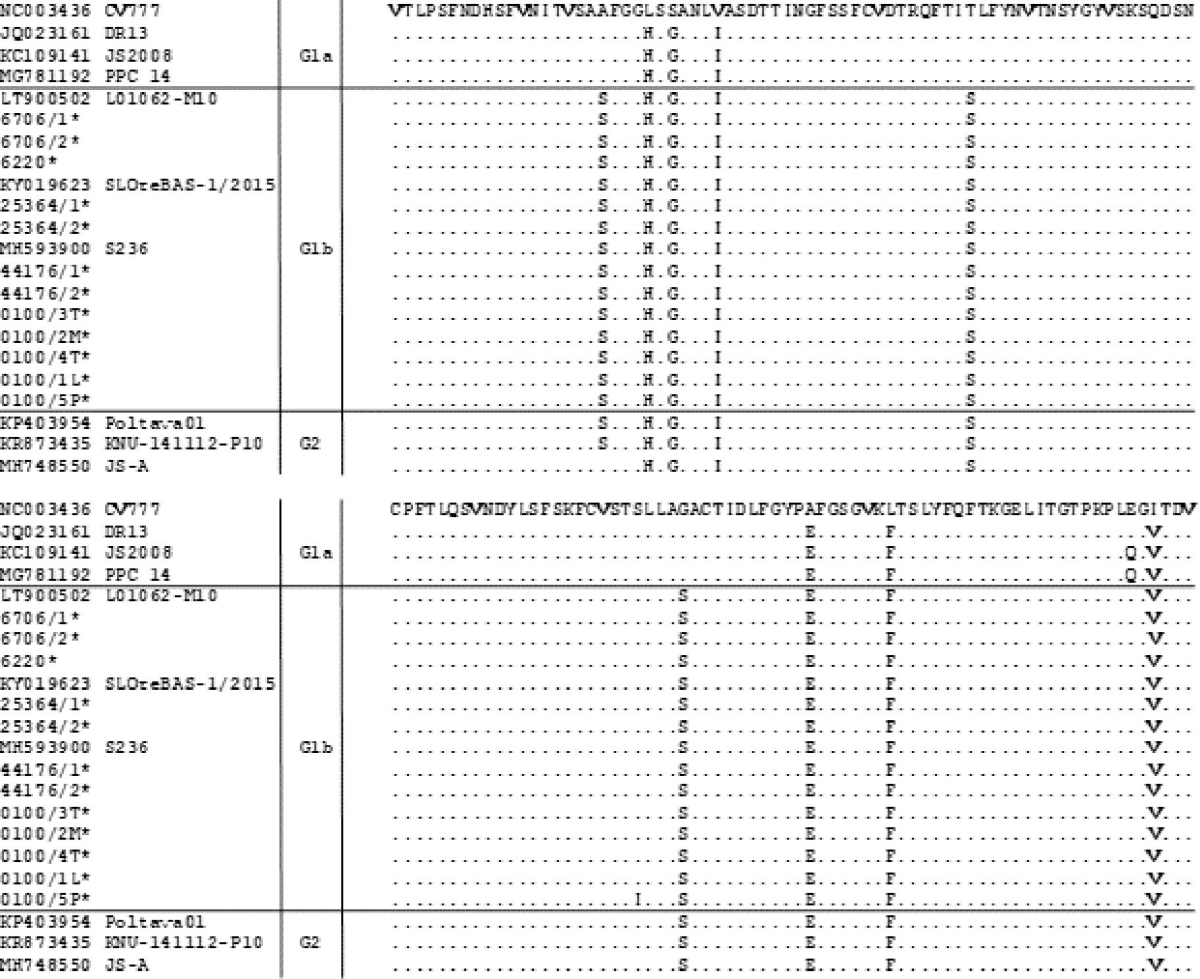
Amino acid alignment of the neutralizing epitope COE located in the S protein of the Polish PEDV strains and reference strains. The dots (.) represent amino acids that are identical. Alignment was constructed using the Clustal W method. Stars indicate the Polish PEDV sequences described in this study.

In order to study the genetic characteristics of the Polish PEDV strains, the deduced amino acid (aa) sequences of the S protein were compared with other reference PEDV strains representing the G2 genogroup (Non-S-INDEL strains) and the G1genogroup (S-INDEL-G1b and classical strains-G1a). We identified all 4 major epitopes capable of inducing neutralizing antibodies, the COE region, YSNIGVCK (SS2), SQSGQVKI (SS6) and GPRLQPY (2C10). In our study, sequences of epitopes SS2, SS6 and 2C10 were well conserved. As shown in Fig 3, compared to classical strain CV777, the Polish PEDV strains had the Y/S substitution at the third position in the SS6 epitope and the R/V substitution at the third position in the 2C10 epitope. Epitope SS2 was almost identical in all analysed strains. In the COE region (Fig 4), the Polish strains had substitutions at position 19 (A/S), 23 (L/H), 25 (S/G), 29 (V/I), 51 (T/S), 96 (G/S), 107 (A/E), 114 (L/F) and 137 (I/V), as compared to strain CV777. The same differences in residues were observed in other S-INDEL strains from the G1b subgroup, as well as in almost all non-INDEL strains belonging to genogroup G2. Furthermore, Polish strain #0100/5P had the unique substitution S/I at the 92 position of the COE epitope.

### Potential recombinant events of the PEDV S gene

**Fig 5:**
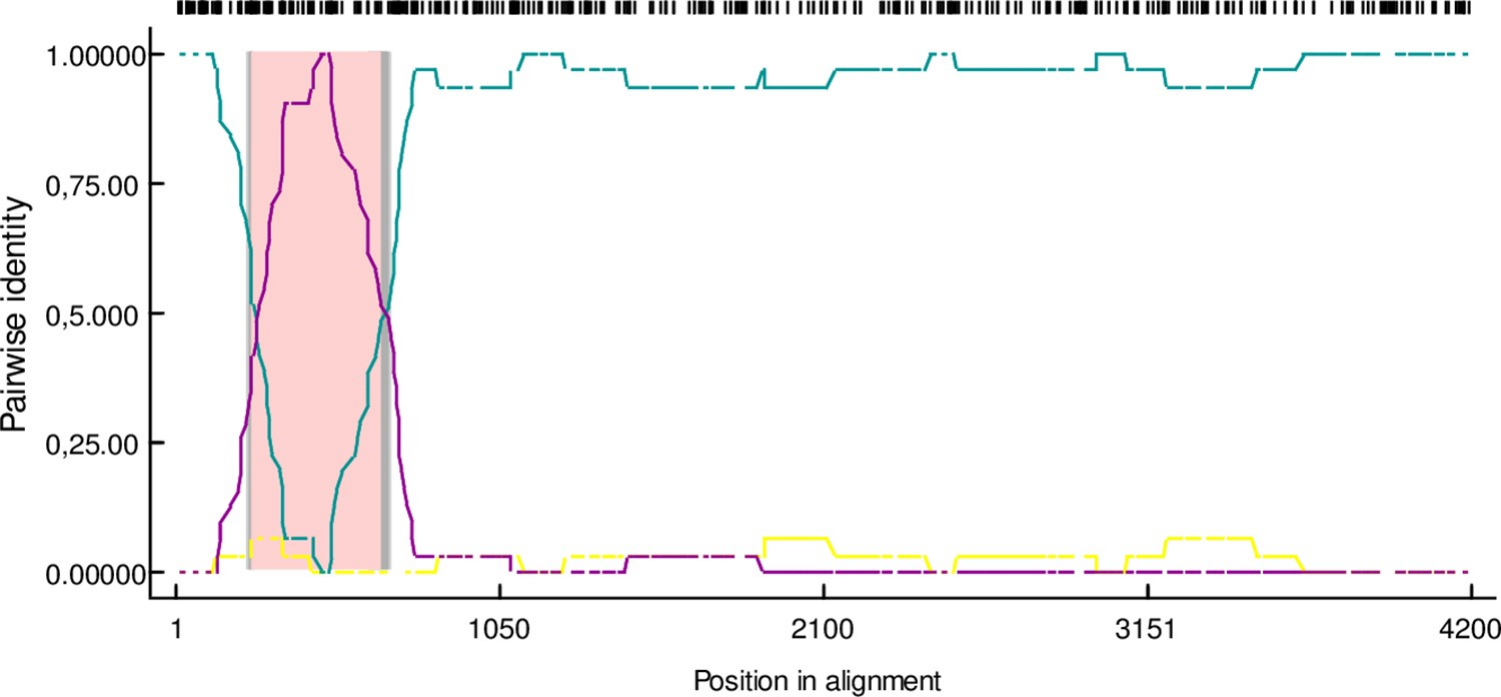
Potential recombination breakpoints in the S gene of the Polish PEDV strains. The potential minor parent was the SeCoV Spanish strain MU2 (MN692770) and the major parent was the recombinant PEDV Spanish strain 1556 (MN692763) or the South Korean strain DR13 (JQ23161). The analysis was performed using the pairwise distance model with a window size of 200, step size of 20 and 1,000 bootstrap replicates by the RPD4 program.

In order to further analyse the association between Polish PEDV strains and those of the existing isolates, a recombination analysis was performed with RDP4 software. In the case of 9 Polish strains (#25364/1, #25364/2, #44176/2, #44176/1, #0100/3T, #0100/4T, #0100/2M, #0100/5P and #0100/1L) belonging to the second and third clusters within the G1b subgroup, all 7 methods assayed consistently detected a recombinant segment located at the 5’ end of the S gene (Fig 5). In this recombination event, the beginning and ending breakpoints were located at the 252 and 672 nucleotides in alignments, the minor parent was the SeCoV Spanish strain MU2 (MN692770) and the major parent was the recombinant PEDV Spanish strain 1556 (MN692763) or the South Korean strain DR13 (JQ23161). Identical recombination events were detected in the Spanish strain H3 (MN692792), the French strain FR2019001 (MN056942), the Slovenian strain SLOreBAS-1/2015 (KY019623) and also in the Hungarian strains: S236 (MH593900) and HUN/5031/2016 (KX289955).

## Discussion

PEDV has become an important diarrhoea virus, causing serious economic losses for the pig industry worldwide. In Poland, the clinical symptoms of PED and the presence of specific antibodies against PEDV have been observed in several farms in 2015–2017 but no studies were performed to confirm the presence of PEDV. This paper, for the first time, presents data concerning the molecular characterization of PEDV strains from Poland.

Based on a phylogenetic analysis, PEDV is mainly divided into two genogroups, G1 (including the G1a and G1b subgroups) and G2 (with the G2a and G2b subgroups) [41, 42]. In this study, a phylogenetic analysis was performed on the basis of whole genome sequences and showed that the Polish PEDV strains belonged to subgroup G1b and closely resembled the European PEDV S-INDEL strains isolated from 2014 to 2019. They were within the range of 99.6%-100% in terms of nucleotide identity. The phylogenetic tree based on the full length S gene, including more sequences of the Polish strains, confirmed the occurrence of the only G1b strain suggesting that this strain may be dominant in Poland. Furthermore, phylogenetic analysis allows for the classification of the Polish PEDV strains into three clusters with some geographical relationships. The first cluster included three strains isolated from 2016 and originated from two distinct farms located in neighbouring voivodeships (Dolnośląskie and Wielkopolskie). The second and third cluster comprised the strains from two herds—also located in neighbouring voivodeships. The second cluster contained two strains from 2015 which originated from one herd located in the Wielkopolskie voivodeship while the isolates identified in this third cluster originated from one herd from Kujawsko-Pomorskie voivodeship. It remains unknown how PEDV was introduced to Poland but positive samples were only detected in 2015–2017, when several emerging PEDV outbreaks were reported in European countries, including Germany [43], Belgium [44], France [45], Italy [46, 47] Austria [48], Portugal [49], Slovenia [50], and Hungary [51], which may suggest that it spread via a transboundary route. In this period of time, an increase in swine imports, as well as pork and pork products to Poland from western Europe (mainly: Germany, Belgium and Denmark) was noted [52, 53]. This situation was correlated with the first detection of African swine fever (ASF) in Poland in 2014 [54] which adversely influenced the recovery of swine production in the following years. The subsequent increased importation of breeding stock from Denmark and Germany to help swine inventories recover and improve genetics [53] could also promote PED introduction to Poland. PEDV is mainly transmitted via the faecal-oral route, but shedding in semen has also been documented [55] and could therefore be a possible source of strains from western Europe, identified in this study. Moreover, indirect transmission via contaminated fomites, as well as cross-contamination via feed cannot be excluded, as was confirmed previously [56, 57].

It has been shown that PEDV S-INDEL strains belonging to the G1b subgroup have a relatively low degree of pathogenicity and cause mild symptoms of the disease compared to the highly virulent non-INDEL strains [1, 17, 58]. For this reason, infection with these strains may be overlooked which additionally complicates the accurate estimation of PED prevalence in Poland. During the epidemic in Asia and America the presence of both genotypes have been confirmed, while in Europe the presence of the non-INDEL variant was only identified in the Ukraine [59]. In other European countries only the occurrence of a variant with a relatively low degree of pathogenicity (S-INDEL) was recorded [17, 24, 55]. However, because PED is not a significant disease in the EU and it is not amongst the World Organisation for Animal Health (OIE) listed diseases, to date most countries have not implemented active monitoring for this particular disease, so the information concerning the currently circulating PEDV variants is limited or unknown, facilitating the subsequent spread of the virus. Moreover, the appearance of further genetic variations should be expected as PEDV strains are especially prone to mutations and recombinations [4, 60, 61].

The S gene is commonly used as the target gene in studies concerning the genomic characteristics of PEDV strains. The PEDV S protein is responsible for receptor binding and viral entry, and thus, determines the host range and cell tropism [62, 63]. In addition, neutralizing epitopes have been found in the S protein thus this protein is a primary target for vaccination against PEDV [4, 64]. Neutralizing antibodies play an important role in the prevention and control of viral infection therefore it is important to analyse changes in their amino acid sequences. Four neutralizing epitopes in the PEDV S protein have been determined, COE [65], 2C10 [66], SS2 and SS6 [67], and all 4 were detected in sequences of the S gene of the Polish PEDV strains. An antigenic analysis showed that the aa sequences of all epitopes were conserved among the Polish PEDV strains. Only one strain, #0100/5P, had a unique substitution in the COE epitope. However, the Polish PEDV strains showed several substitutions, especially in the COE antigen, as compared to the classical strain CV777. The same differences in residues were observed in other S-INDEL strains from the G1b subgroup as well as in most of the non-INDEL strains belonging to genogroup G2. Mutations in the sequences of epitopes may alter the antigenicity, pathogenicity and neutralization properties of strains [68]. Therefore, a vaccine derived from the prototype strain CV777 protects against the disease caused by classical strains [69] but does not provide adequate immune protection against the currently emerging strains [58, 70–72]. Recent vaccine strains may only partially induce neutralizing antibodies against emergent PEDV strains which pose a major challenge to the prevention and control of PED.

Recombination plays a pivotal role in the diversity and evolution of coronaviruses by creating new strains with altered virulence [73]. Several reports have identified recombinant sequences in the PEDV S gene, ORF1a and ORF1b [69, 74]. Since 9 of the Polish PEDV strains are clustered together with recombinant PEDV-SeCoV isolates described in Hungary, Italy, Spain and Slovenia [24, 51, 75, 76] we verified whether or not Polish PEDV strains also resulted from such a recombination event. RDP4 analysis confirmed that Polish PEDV strains harbour a recombinant fragment of ~400 nt in the 5’ end of the S gene with PEDV and SeCoV being the major and minor parents, respectively. Putative recombination events were detected using all 7 statistical methods with a high degree of significance and reliability. Our results indicated that recombination is a common phenomenon among Polish field PEDV strains since 9 out of 12 of the analysed strains represented a recombinant PEDV-SeCoV variant. The clear separation of the PEDV-SeCoV isolates from the other European strains, suggests a new independent evolution of PEDV in Europe from 2015 due to a recombination event in the S-gene between PEDV and SeCoV. Detection of new chimeric (recombinant) coronavirus affecting pigs called swine enteric coronavirus (SeCoV) should clarify the origin of the novel recombinant PEDV isolates. Most of the genome of this new chimeric virus is derived from TGEV, but the S-gene is derived from PEDV [75]. SeCoV was reported in several European countries including Italy [75], Germany [77], Spain [de Nova] and countries from Central Eastern Europe [78, 79]. It causes the same clinical signs as PEDV and TGEV and because their recombinant nature, a diagnosis based on the detection of a particular sequence for both PEDV and TGEV may lead to misidentification and the presence of SeCoV may be unnoticed [de Nova]. In Poland, no studies have been performed to confirm the presence of SeCoV.

In conclusion, the Polish PEDV strains identified in this study clustered into a G1b subgroup and were closely related to the European PEDV S-INDEL strains. Compared with the prototype strain CV777, Polish PEDV strains had multiple variation in neutralizing epitopes, suggesting that the development of a novel vaccine may be necessary for the control of PED in Poland. Furthermore, in this study, a natural recombination event involving a ~ 400 nt fragment of SeCoV was identified in the Polish PEDV strain. To the best of authors’ knowledge, this is the first report concerning the genetic characteristics of the virus in Poland. These results provide valuable information concerning PEDV strains circulating in the country which is especially important for the effective control of the disease and limiting the losses in swine production.

## Supporting information

S1 Fig Phylogenetic relationship between the sequences of PEDV Polish strains and sequences of reference strains obtained from GenBank. The phylogenetic tree was constructed on the basis of the sequences of I fragment of S gene with MEGA6 software using neighbor-joining method. Bootstrap value >70 are shown. The numbers of each branch represent the bootstrap value calculated by 1000 replicates. The scale bars indicate nucleotide substitutions per site. The S gene sequences from PEDV isolates identified in this study are indicated with filled black circles.
(TIF)

S2 Fig Phylogenetic relationship between the sequences of PEDV Polish strains and sequences of reference strains obtained from GenBank. The phylogenetic tree was constructed on the basis of the sequences of II fragment of S gene with MEGA6 software using neighbor-joining method. Bootstrap value >70 are shown. The numbers of each branch represent the bootstrap value calculated by 1000 replicates. The scale bars indicate nucleotide substitutions per site. The S gene sequences from PEDV isolates identified in this study are indicated with filled black circles.
(TIF)

S3 Fig Phylogenetic relationship between the sequences of PEDV Polish strains and sequences of reference strains obtained from GenBank. The phylogenetic tree was constructed on the basis of the sequences of III fragment of S gene with MEGA6 software using neighbor-joining method. Bootstrap value >70 are shown. The numbers of each branch represent the bootstrap value calculated by 1000 replicates. The scale bars indicate nucleotide substitutions per site. The S gene sequences from PEDV isolates identified in this study are indicated with filled black circles.
(TIF)

S4 Fig Phylogenetic relationship between the sequences of PEDV Polish strains and sequences of reference strains obtained from GenBank. The phylogenetic tree was constructed on the basis of the sequences of IV fragment of S gene with MEGA6 software using neighbor-joining method. Bootstrap value >70 are shown. The numbers of each branch represent the bootstrap value calculated by 1000 replicates. The scale bars indicate nucleotide substitutions per site. The S gene sequences from PEDV isolates identified in this study are indicated with filled black circles.
(TIF)
