# Correction: Molecular characterization of porcine epidemic diarrhoea virus (PEDV) in Poland reveals the presence of swine enteric coronavirus (SeCoV) sequence in S gene

**DOI:** 10.1371/journal.pone.0315018

**Published:** 2024-12-02

**Authors:** Marta Antas, Monika Olech, Anna Szczotka-Bochniarz

The ORCID iD of the third author is missing. Please see Anna Szczotka-Bochniarz’s ORCID iD here: https://orcid.org/0000-0001-8422-6848
