# Supplementary figures and images for: Molecular characterization of porcine epidemic diarrhoea virus (PEDV) in Poland reveals the presence of swine enteric coronavirus (SeCoV) sequence in S gene
Source: PLoS One. 2021 Oct 29;16(10):e0258318. doi: 10.1371/journal.pone.0258318 (PMC8555794; doi:10.1371/journal.pone.0258318)

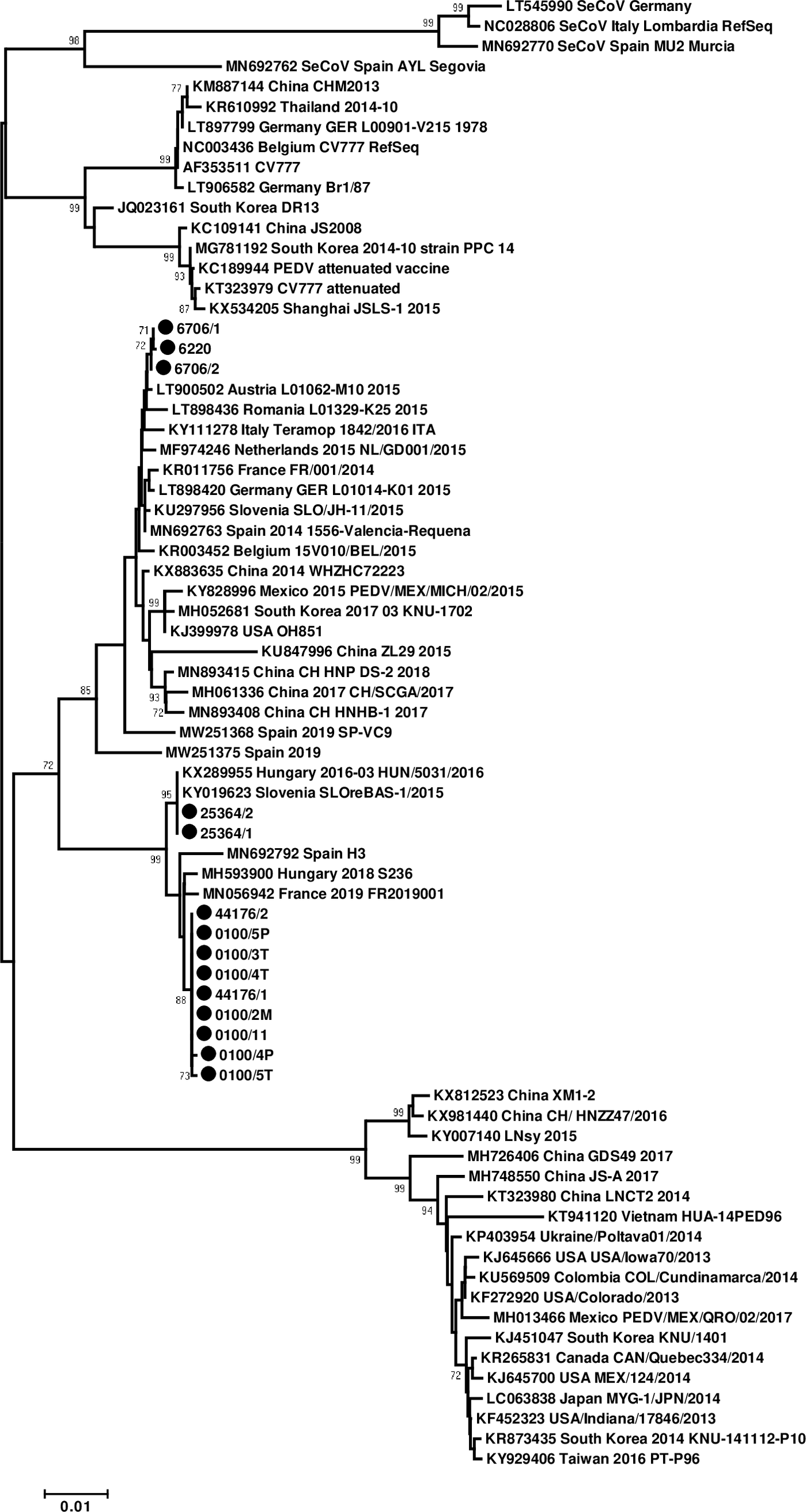

Supplement: S1 Fig — The phylogenetic tree was constructed on the basis of the sequences of I fragment of S gene with MEGA6 software using neighbor-joining method. Bootstrap value >70 are shown. The numbers of each branch represent the bootstrap value calculated by 1000 replicates. The scale bars indicate nucleotide substitutions per site. The S gene sequences from PEDV isolates identified in this study are indicated with filled black circles. (TIF) [file pone.0258318.s001.tif]

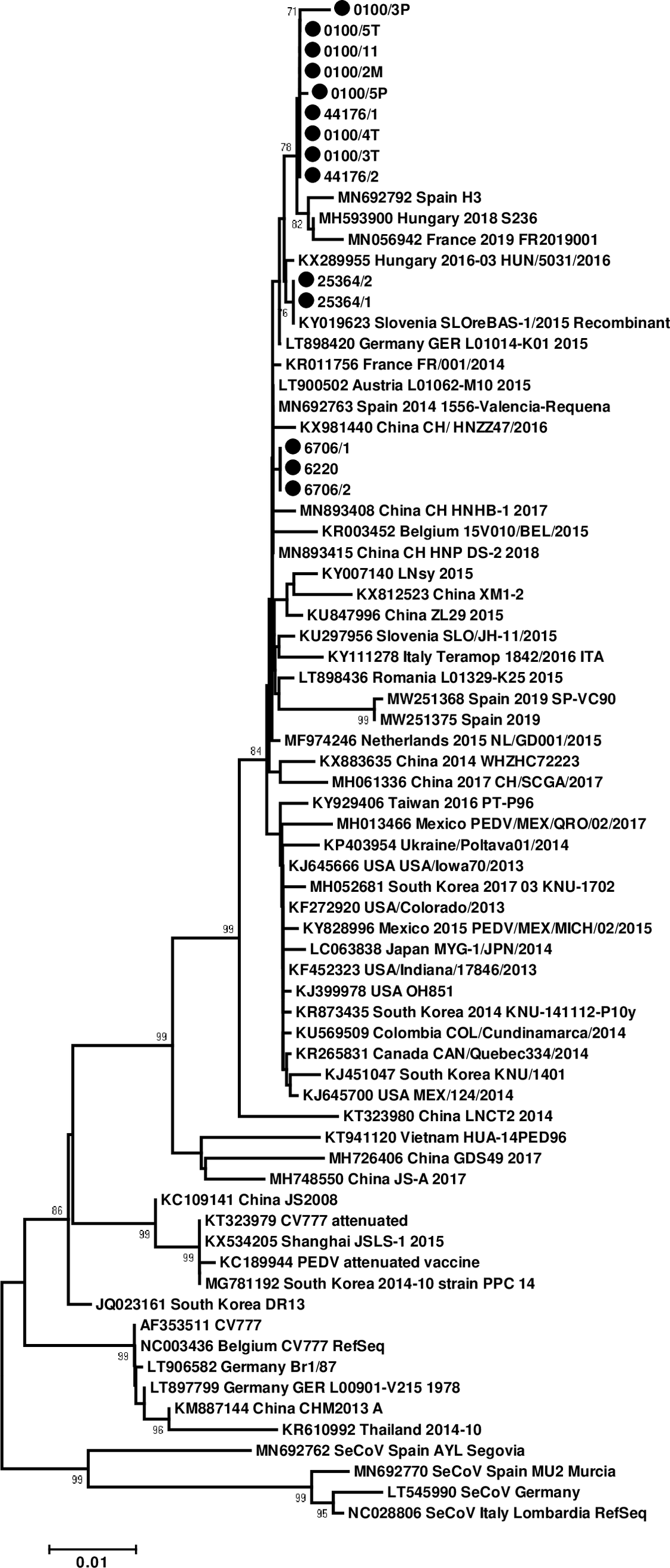

Supplement: S2 Fig — The phylogenetic tree was constructed on the basis of the sequences of II fragment of S gene with MEGA6 software using neighbor-joining method. Bootstrap value >70 are shown. The numbers of each branch represent the bootstrap value calculated by 1000 replicates. The scale bars indicate nucleotide substitutions per site. The S gene sequences from PEDV isolates identified in this study are indicated with filled black circles. (TIF) [file pone.0258318.s002.tif]

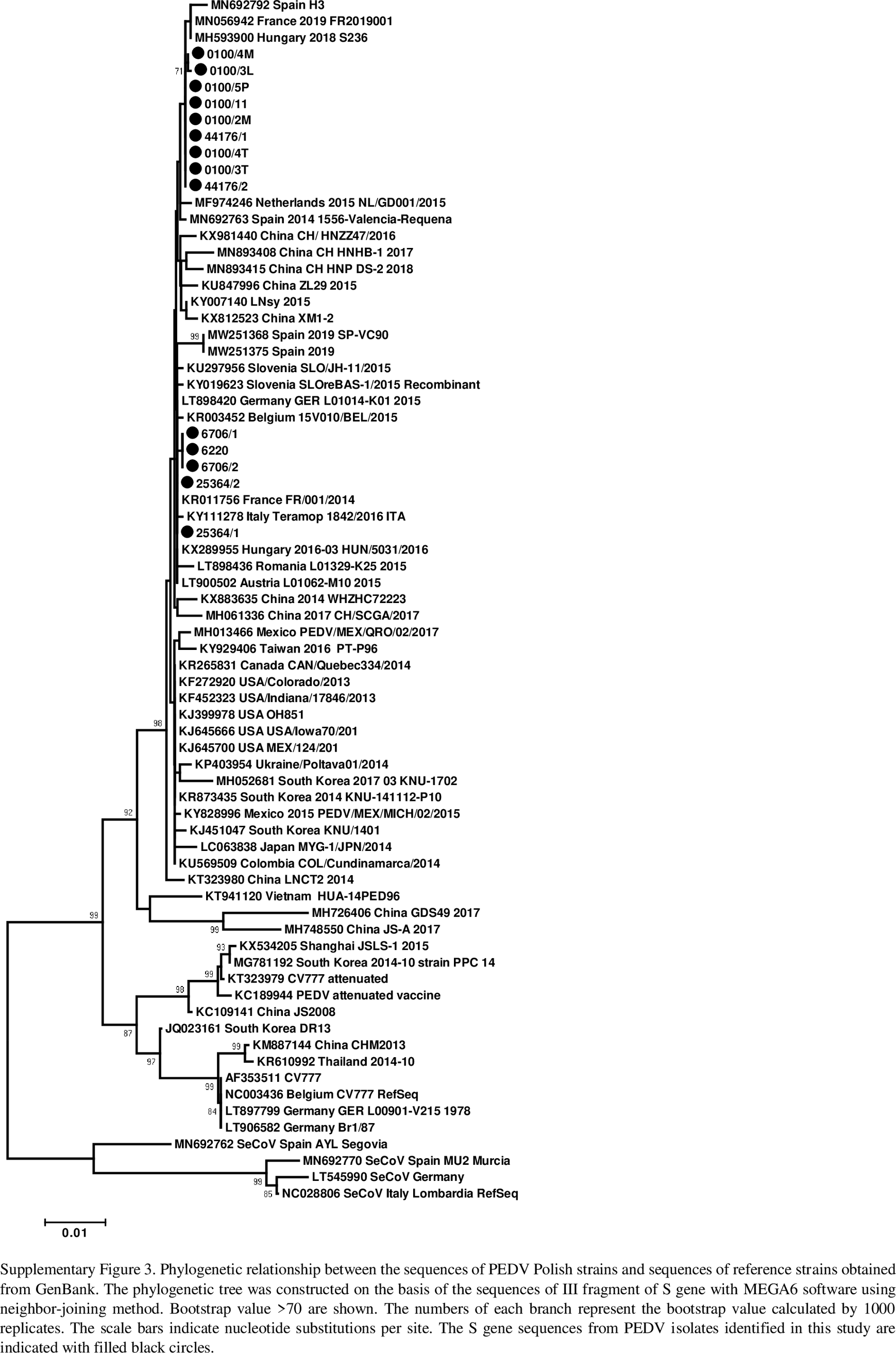

Supplement: S3 Fig — The phylogenetic tree was constructed on the basis of the sequences of III fragment of S gene with MEGA6 software using neighbor-joining method. Bootstrap value >70 are shown. The numbers of each branch represent the bootstrap value calculated by 1000 replicates. The scale bars indicate nucleotide substitutions per site. The S gene sequences from PEDV isolates identified in this study are indicated with filled black circles. (TIF) [file pone.0258318.s003.tif]

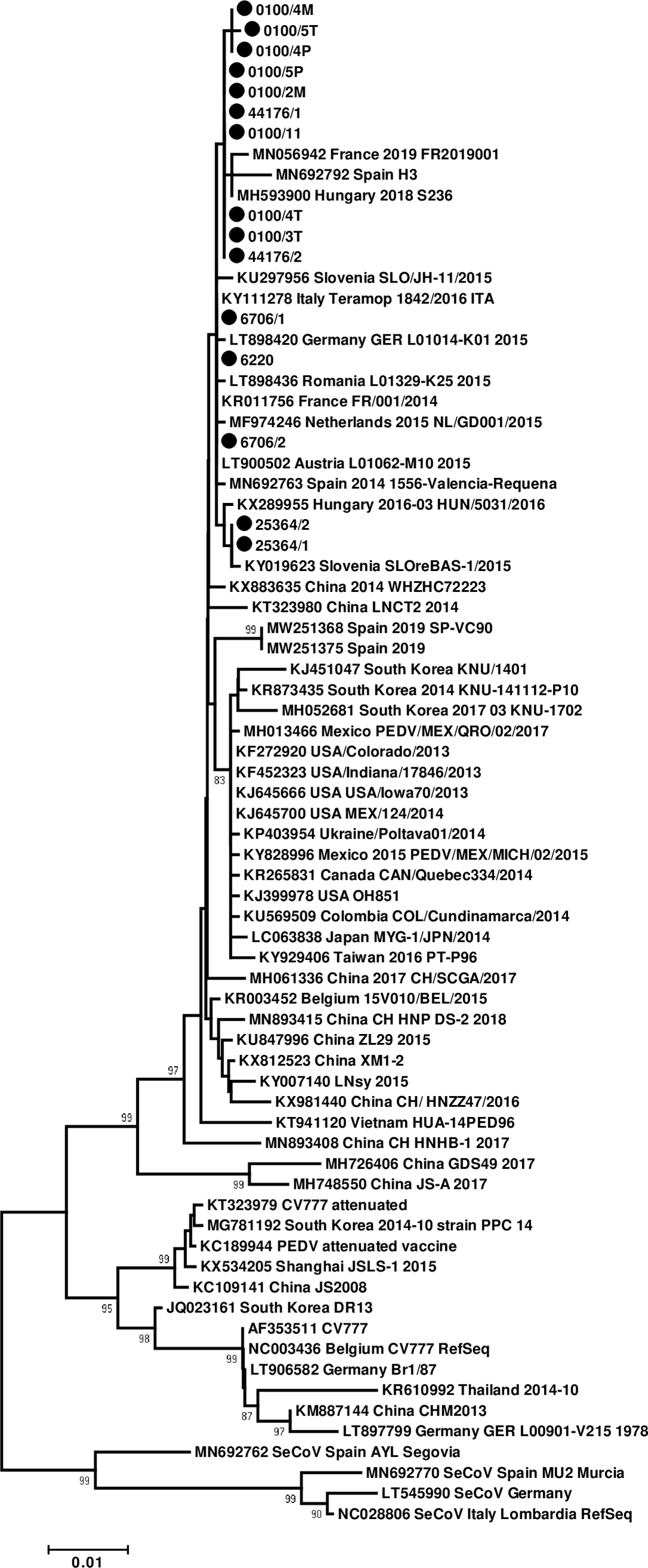

Supplement: S4 Fig — The phylogenetic tree was constructed on the basis of the sequences of IV fragment of S gene with MEGA6 software using neighbor-joining method. Bootstrap value >70 are shown. The numbers of each branch represent the bootstrap value calculated by 1000 replicates. The scale bars indicate nucleotide substitutions per site. The S gene sequences from PEDV isolates identified in this study are indicated with filled black circles. (TIF) [file pone.0258318.s004.tif]
